# Response Surface Methodology (RSM) Powered Formulation Development, Optimization and Evaluation of Thiolated Based Mucoadhesive Nanocrystals for Local Delivery of Simvastatin

**DOI:** 10.3390/polym14235184

**Published:** 2022-11-28

**Authors:** Rana B. Bakhaidar, Nimbagal Raghavendra Naveen, Pratap Basim, Samar S. Murshid, Mallesh Kurakula, Abdulmohsin J. Alamoudi, Deena M. Bukhary, Abdulmajeed M. Jali, Mohammed A. Majrashi, Sameer Alshehri, Mohammed Alissa, Rayan A. Ahmed

**Affiliations:** 1 Department of Pharmaceutics, Faculty of Pharmacy, King Abdulaziz University, Jeddah 21589, Saudi Arabia; 2 Sri Adichunchanagiri College of Pharmacy, Adichunchanagiri University, B. G. Nagar, Karnataka 571448, India; 3 Thermo Fisher Scientific, Cincinnati, OH 45237, USA; 4 Department of Natural Products and Alternative Medicine, Faculty of Pharmacy, King Abdulaziz University, Jeddah 21589, Saudi Arabia; 5 Thermo Fisher Scientific, Bend, OR 97701, USA; 6 Department of Pharmacology and Toxicology, Faculty of Pharmacy, King Abdulaziz University, Jeddah 21589, Saudi Arabia; 7 Department of Pharmaceutics, College of Pharmacy, Umm Al-Qura University, Makkah 24381, Saudi Arabia; 8 Department of Pharmacology and Toxicology, College of Pharmacy, Jazan University, Jazan 45142, Saudi Arabia; 9 Department of Pharmacology, College of Medicine, University of Jeddah, Jeddah 23890, Saudi Arabia; 10 Department of Pharmaceutics and Industrial Pharmacy, College of Pharmacy, Taif University, Taif 21944, Saudi Arabia; 11 Department of Medical Laboratory Sciences, College of Applied Medical Sciences, Prince Sattam bin Abdulaziz University, Al-Kharj 11942, Saudi Arabia

**Keywords:** health care, simvastatin, xanthan gum, thiolation, mucoadhesion, response surface methodology

## Abstract

In oral administration systems, mucoadhesive polymers are crucial for drug localization and target-specific activities. The current work focuses on the application of thiolated xanthan gum (TXG) to develop and characterize a novel mucoadhesive nanocrystal (NC) system of simvastatin (SIM). Preparation of SIM-NC was optimized using response surface methodology (RSM) coupled with statistical applications. The concentration of Pluronic F-127 and vacuum pressure were optimized by central composite design. Based on this desirable approach, the prerequisites of the optimum formulation can be achieved by a formulation having 92.568 mg of F-127 and 77.85 mbar vacuum pressure to result in EE of 88.8747% and PS of 0.137.835 nm. An optimized formulation was prepared with the above conditions along with xanthan gum (XG) and TXG and various parameters were evaluated. A formulation containing TXG showed 98.25% of SIM at the end of 96 h. Regarding the mucoadhesion potential evaluated by measuring zeta potential, TXG-SIM-NC shoed the maximum zeta potential of 16,455.8 ± 869 mV at the end of 6 h. The cell viability percentage of TXG-SIM-NC (52.54 ± 3.4% with concentration of 50 µg/mL) was less than the plain SIM, with XG-SIM-NC showing the highest cytotoxicity on HSC-3 cells. In vivo pharmacokinetic studies confirm the enhanced bioavailability of formulated mucoadhesive systems of SIM-NC, with TXG-SIM-NC exhibiting the maximum.

## 1. Introduction

Nanomedicines have opened up new opportunities for therapeutic treatments because biocompatible nanosize-based particles may enhance the overall pharmacological properties of a given drug substance, including improved bioavailability [1] and targeting, extended release, decreased side effects [2,3,4], and expanding various administration routes [5]. Thanks to recent advancements in nanotechnology, it is now possible to create drug carriers with nanometer-scale features, such as nanoparticles, that can transport medications to particular places. In order to provide safe and effective formulations for diverse ailments, the development of drug delivery technology has led to the employment of several approaches, including surfactants, inclusion complexation, and solid dispersion [6]. Notably, drug molecules crystallize to form drug nanocrystals (NCs), which are pure solid drug particles with diameters between 10 and 1000 nm that are encased in a stabilizer layer [7,8]. Due to their large specific surface area and minimal addition of surfactants as stabilizers, they are a colloidal dispersion system with higher saturation solubility and drug loading [9]. Drug NCs, which combine essential stabilizers and active pharmaceutical ingredients, can be administered orally, intravenously, or through other routes and have the potential to enhance the solubility, dissolution, and bioavailability of drugs that are poorly water-soluble or potential new drug molecules [10,11].

Globally, there were 19.3 million new cancer diagnoses and 10.0 million cancer deaths in 2020, estimated by the Global Cancer Observatory (GLOBOCAN). In addition to being the leading cause of death worldwide, cancer also makes it challenging to increase life expectancy across the board [12]. Chemotherapy is often used to treat cancer, but it has drawbacks such as low solubility, limited targeting ability, and toxicity that lead to insufficient drug enrichment at the tumor site, limiting its clinical efficacy [13,14].

Due to the weak and heterogeneous enhanced permeability and retention (EPR) effect in solid tumors in the context of human tumors, the use of commercial nanomedicines for chemotherapy based on passive targeting, such as drug-loaded liposomes, micelles, and nanoparticles, has encountered a bottleneck [15]. Drug delivery to tumors has long utilized passive targeting. Highly effective medication delivery at the tumor site is gaining attention in order to address the limitations of the passive targeting strategy that are present in practical applications, such as non-specific drug distribution, excessive administration dosage, and undesired side effects [16]. Continuous advancements in active tumor-targeted pharmaceutical NC delivery systems have been made in recent years, and most of these initiatives are encouraging and instructional for future study and clinical application.

Natural gums and mucilage of plants are impervious as a pharmaceutical excipient, particularly in the formulation of controlled drug forms [17]. These substances’ physical and chemical characteristics can easily be modified to achieve the requirements of an ideal drug delivery system [18]. Xanthan gum is a natural, high-MW polysaccharide obtained by the fermentation of sugars with Xanthomonas campestris bacteria (usually present on the leaves of green vegetables, especially in the cabbage family) [19]. It was studied extensively for pharma, cosmetic, and food applications as an excipient, stabilizing agent, viscosity enhancer, hardening agent, and emulsifying and suspending agent.

Thiolation of the mucoadhesive polymers provides the potential to make disulfide bonds (inter-/intrachain) within the polymeric system and can significantly enhance their cohesive nature. The chemical reaction of thiol moiety with mucin-containing cysteine results in the development of strong covalent bonds [20,21]. Thiomers, in contrast to unaltered polymers, exhibit good adhesive properties that are adequate to restrain the drug at required target sites for a longer duration. In addition, thiolated polymers have the effects of enzyme inhibition, improved penetration, controlled release, and thermal stability [22].

Simvastatin (SIM) is significantly metabolized by microsomal enzymes and has a lower bioavailability (5%). SIM is a biopharmaceutics chemical that falls under the Biopharmaceutics Classification System (BCS) Class-II category. It has low aqueous solubility and a good permeability through biomembranes. The cytochrome enzyme CYP3A4 primarily targets the lactone structure of SIM and significantly reduces intestinal absorption. Low bioavailability is caused by the medicine’s aquaphobic character, which prevents complete drug dissolution in the intestinal medium. Statins are generally effective in maintaining cholesterol levels and lower blood cholesterol by inhibiting 3-hydroxy-3-methyl glutaryl coenzyme A (HMG-CoA) [22,23]. SIM was acknowledged for its potential to treat several malignancies by preventing metastasis, inducing apoptosis, and slowing down the cell cycle [24]. Ras and other small G proteins are altered in their prenylation due to HMG-CoA reductase inhibition, which affects the downstream signaling pathways that control cell growth and survival. As a result, statins’ inhibition of HMG-CoA reductase was discovered to cause apoptosis in several cancer cells. Recent research by Masashi et al. demonstrated that statins reduced the activation of the Ras/ERK1/2 and Ras/phosphoinositide 3-kinase/Akt pathways. Statins cause apoptosis in malignant glioma cells either by activating JNK1/2 or by upregulating the expression of Bim [25].

The response surface methodology (RSM) is a group of statistical methods used to create and assess the relationship between a response and a set of relevant variables. It is possible to study the optimization process using the data gathered in this way from an experiment [26]. RSM is significantly more efficient and cost-effective than traditional formulation development approaches since it involves less testing and time [27,28,29]. In order to identify the best formulation(s), RSM revolves around the generation of polynomial equations and response over the experimental domain. A minimal number of experiments are needed to effectively estimate the impact of individual variables and examine their interactions when employing a factorial design. In this study, we investigated the application of thiolated xanthan gum (TXG) to improve SIM’s nanocrystal formulation’s mucoadhesive properties for enhanced local retention in the upper part of the gastrointestinal track, to enhance the absorption and bioavailability of SIM. In this study, SIM was selected as a model drug to target the cancer cells of GIT. Our previous research paper [19] stated that TXG was created by thiol esterifying XG with thioglycolic acid. We believe that this is the first study to examine how TXG affects SIM-NC features, particularly its mucoadhesive properties.

## 2. Materials and Methods

### 2.1. Materials

Biocon Pvt Ltd., Bangalore, India generously provided SIM. Pluronic F-127, chloroform, dextrose and xanthan gum were procured from Loba Chemie Pvt Ltd., Mumbai, India. All other chemicals used were of analytical grade.

### 2.2. Preparation of SIM-NCs

The NCs were prepared using a three-phase nanoparticle engineering technique (3PNET), which contains phases 1 and 2 of hydrated amorphous aggregate, phase 3 of stabilized NCs, and phase 1 of amorphous precipitate [30]. In a nutshell, 40 mg of SIM and Pluronic F-127 were first dissolved in chloroform (in a glass tube) at various weight ratios, and then the chloroform was evaporated using a constant stream of nitrogen gas to produce coprecipitation. The solubilized drug was precipitated by rota vaporizing the entire solution to a pressure of 40–80 mbar, maintaining that pressure for 30 min, then lowering it to 10 mbar. The remaining chloroform was eliminated under a vacuum with desiccators for 2–4 h. After 1 h of hydration (in 1 mL of 5% dextrose) and vortexing, the suspensions were sonicated for 10 to 15 min using an 80 kc, 80 W bath-type sonicator while adding XG and TXG to create the final SIM-NCs.

### 2.3. Experimental Design

The statistical model RSM standardized the synthesis of SIM-NC. The F-127 (X_1_) and vacuum pressure (X_2_) were selected as independent parameters at five different values and are coded as −1.414 (low), −1, 0 (medium), +1, and +1 (high) [31,32,33] (Table 1). Using Design Expert Version 12 (Stat Ease Inc., Minneapolis, MN, USA), 13 experiment runs were created to examine these factors’ effects on the size of nanocrystals (PS) and entrapment efficacy (EE) and a variety of statistical approaches were used to choose the model that suited the data the best. Each test run used a quadratic design to quantify the response and regression analysis.

#### 2.3.1. PS

Using the dynamic light scattering technique, a Malvern Zetasizer-2000 (Cambridge, UK) analyzed the SIM-NC average PS, PDI, and electrokinetic potential. To prevent the blockage of particles, the prescribed amount of SIM-NC was re-dispersed into a generous amount of Milli-Q water and vortexed for 5 min. At 25 °C, the final sample was evaluated in triplicate for 1 min [34].

#### 2.3.2. EE

The NCs were kept at room temperature for 1 h, passed through 0.45 and 0.22 mm centrifugal filters and a 3000 MWCO microcon (Millipore Co., Bedford, MA, USA), and centrifuged at 16,000× *g* for 20 min to test SIM encapsulation [35]. A water bath was used to combine and drain the cleaning materials and supernatant fluid, and the resulting mixture was then diluted with methyl alcohol. SIM’s absorbency was determined at 450 nm. In comparison to a theoretical amount, EE was calculated.

### 2.4. Standardization and Validation of Optimization Outcome

Software called Design-Expert was used to trigger the reactions that all of the preparations had to provide. The study technique and the response surface graph were developed using the responses. A numerical standardization method created an optimal formula with the lowest and maximal limits for each parameter. A desirability function was created by incorporating the results. The answers that satisfied the criteria were documented, and the set of options was ranked in order of desirability. The response surface graph clarified the relationship between the independent and dependent parameters. ANOVA was used to examine how different variables affected the slope coefficients [36]. The difference between predicted and experimental values was used to calculate the relative uncertainty as part of the design validation process. With the circumstances described by Design-Expert, an optimized formulation (O-SIM-NC) of SIM-NC was created and tested in various in vitro and in vivo settings. Two formulations using XG and TXG were intended to compare the mucoadhesion potential of created formulations.

### 2.5. SIM-NC Morphology

A scanning electron microscope (SEM) (Philips XL 30 microscope, Hillsboro, OH, USA) was used to examine NCs’ morphology. Raw ETO MC powder and processed NC powder were applied to a double-sided tape, coated with a 30 nm coating of gold, and then subjected to a 2 min period of vacuum (10-6 Pa) and SEM observations at a 15 kV accelerating voltage [37].

#### X-Ray Diffraction (XRD)

An analytical method known as XRD is quick and is generally used to determine the phase of crystalline materials. Crystallinity of pure SIM and SIM-NC formulations was investigated utilizing XRD analysis using Inxitu Benchtop XRD (Mountain View, CA, USA).

### 2.6. Drug Release Study

To examine release kinetics, 10,000 Da dialysis cassettes were filled with 1 mL of O-SIM-NC and submerged in 400 mL of dialysis buffer (PBS (pH 7.4) containing 0.05% Tween 80). At particular times, 2 mL aliquots of buffer were UV spectroscopically measured [38]. To maintain a sink condition, the entire volume of the dialysis buffer was replenished at predetermined intervals. Three copies of the study were carried out, and the mean results were recorded.

### 2.7. Mucoadhesive Evaluation of SIM-NC: Zeta Potential Determination and Turbidimetric Measurement

The mucoadhesive assessment of both SIM-NC formulations was evaluated in vitro using two different techniques [39,40]. In the first technique, the mucoadhesive qualities of SIM-NC were assessed by tracking zeta potential changes after contact with negatively charged mucin. The NCs were incubated in a mucin dispersion of 0.1% at 37 °C. During incubation, the zeta potential of the NCs was monitored for up to 4 h. The NCs’ altered zeta potential suggests that mucin was involved in the interaction. Using an ultraviolet–visible spectrophotometer, turbidimetric measurements of SIM-NC were compared with mucin dispersion at 650 nm. Aqueous mucin dispersion (5 mL) and correctly sampled SIM-NC (5 mL) were combined, and the mixture was agitated at 200 rpm. At specific time intervals, the turbidity of the dispersions was measured and contrasted with the turbidity of the mucin dispersion. The mucoadhesive capability of mucin NCs was shown by an increase in turbidity [41].

### 2.8. In Vitro Cell Viability Assay

Using an in vitro model and the MTT assay, the cytotoxicity of the standardized formulation (O-SIM-CAN) was assessed in HCS-3 cells (3-[4,5-dimethylthiazol-2-yl]-2,5-diphenyltetrazolium bromide). HCS-3 cells were trypsinized using 0.25% trypsin-EDTA and put in 96-well plates (about 2500–5000 cells/well) at a density of 5 104 cells/mL. The entire medium was then replaced with Dulbecco’s modified Eagle medium (DMEM) (Sigma Aldrich, Bangalore, India) after 24 h. The absolute test samples were given to the other cells in the range of 10–50 g/mL, whereas 5-fluorouracil was administered to the reference cells. After 72 h of incubation, 0.1 mL of DMEM containing 0.2 mg/mL MTT was added, and the mixture was incubated for an additional 2–3 h. DMSO was used in place of DMEM to eliminate the developed formazan. A microplate reader (Biotek Synergy, Santa Clara, CA, USA) was used later to measure the absorbance at 540 nm. IC 50 values were calculated when the dose–response relationship was established [42].

### 2.9. In Vivo Pharmacokinetic Studies

In vivo studies were conducted after obtaining approval from the Animal Ethical Committee of the Institution of the Clinical Laboratory Center, Beni Suef, Egypt (Approval no. 22/9-09-22). PK solver software was used to analyze the pharmacokinetic (PK) performance of NC after oral and transdermal delivery. Wistar albino adult male rats weighing approximately 180–250 g were used. The following is a breakdown of a single dose trial of four groups of six animals each. Simvastatin suspension (10 mg/kg) was used in Test Group I. XG-SIM-NC (10 mg/kg) and XG-SIM-NC (10 mg/kg) were used in Test Groups II and III.

Prior to receiving medication formulations, animals fasted for 24 h while still having unrestricted access to water. The abdominal hair was cut the day before the experiment by using a depilatory product for 10 min and then washing it with distilled water. The animals were sedated with ketamine (10 m/kg, i.p.) and placed in a supine position on the day of the experiment. With the aid of an oral feeding needle, all test samples were given orally (10 mg/kg). A retro-orbital puncture was used to collect blood samples totaling roughly 0.5 mL at intervals of 0.5, 2, 4, 8, 12, 24, and 48 h following oral administration. Capillary tubes were used to transfer the samples from a retro-orbital puncture into a glass tube that had been heparinized and included the anticoagulant ammonium oxalate (1% solution). The plasma was promptly separated by microcentrifugation at 5000 rpm and then kept at 20 °C pending HPLC analysis [43,44].

#### 2.9.1. Sample Preparation and Simvastatin Medication Concentration Measurement

After centrifuging 1.5 mL of animal blood at 5000 rpm for 5 min, 0.75 mL of plasma was recovered. Then, 0.5% trichloroacetic acid was added to this sample. Medicine was separated from plasma at 4000 rpm for 15 min at 4 °C [45,46]. The simvastatin in the plasma sample was measured using the supernatant solution put into HPLC. HPLC measured plasma simvastatin levels. Flow rate: 1.0 mL/min; injection volume: 5 uL; column: reversed-phase C18 column (250 mm 4.6 mm i.d., 5 m particle size).

#### 2.9.2. Analyzing Pharmacokinetics

The PK solver application shows HPLC data on time versus plasma drug concentration. Peak plasma concentration (Cmax), time required to attain Cmax (tmax), area under the curve (AUC0-t), and (AUC0-) were directly read from the individual plasma drug concentration versus time profile. Biological half-life (t1/2) and mean residence time (MRT) were also calculated using PK solver software [47].

## 3. Results and Discussion

The effect of particular variables and how they interacted to produce the minimal PS and greatest EE were studied using CCD. Thirteen experimental trials in total were anticipated, and Table 2 lists the observed results. Experimental formulations’ PS was between 130 and 315 nm. EE, which determines how much medication is entrapped, ranged from 62 to 94%. The fx model and ANOVA analyzed the experimental results for particular reactions.

The sequential sum of squares (Type-I) and fit summary were used to pick the quadratic model for all responses. When choosing the models, *F*-value, *p*-value, and *R*^2^ values were considered. The largest polynomial order is seen in the quadratic model, with a *p*-value (degree of significance) of 0.0001 (Table 3).

For EE, the difference between the predicted *R*^2^ of 0.8618 and the adjusted *R*^2^ of 0.9603 is less than 0.2. Adequate precision measures the ratio of signal to noise. A ratio of at least 4 is preferred. A strong signal is indicated by the ratio of 51.052. To move around the design space, this model was utilized. Similar outcomes were seen for PS (0.8174, 0.9450, and 18.7863) [33].

The accuracy of all these selected models was further confirmed by the normal plot of residuals [48]. For this, the recommended statistical application was not used because the visual inspection graph was sufficient. The proposed model can be accepted statistically because all of the studentized residuals for the selected responses were distributed closer to the straight line [26,27]. Figure S1 (Supplementary Materials) represents the experimental run versus the residuals to identify the underlying variables influencing the responses. Within the allowed range, a scattered trend was seen, indicating the presence of a time-coupled variable in the background. The coefficient of variation (CV) value can be used to establish that an experiment’s repeatability which ensures accurate results and transparency in understanding the process. As the required CV value was less than the prescribed value (CV10%) (2.29% for EE and 7.49% for PS), the design’s consistency and accuracy were guaranteed. Lack of fit is an additional parameter that assesses how well the model captures all data. The ANOVA findings clearly show that the lack of fit is non-significant (*p* > 0.05), which supports the fitness of the chosen design. The *p*-values for both responses were determined to be 0.0745 and 0.1115, respectively, indicating a non-significant lack of fit.

An ANOVA was used to investigate the quantitative impacts of particular factors on responses [49,50]. Multiple regression was applied to the collected data to produce polynomial equations. All of the chosen models are likely to be significant, according to the model *F*-values of 214.68, 90.00, and 158.71. [51].

A, B, AB, A^2^, and B^2^ are significant model terms in the case of EE. According to the experimental plan, EE might be impacted by two factors: (i) an antagonistic effect of component B; (ii) a synergistic effect of A and AB, with AB impacts being the more significant; and (iii) polynomial terms of A and B. According to the experimental plan, PS could be impacted by the antagonistic effects of factors A and B as well as the synergistic effects of polynomial term B, with B effects having the most impact. The polynomial term of factor B and its *p*-value are 0.0001 and 0.0132, respectively (Table 4). Using the equation with coded factors, one may predict the reaction for specific element amounts. High levels are coded as +1 and low levels as −1. By comparing factor coefficients, the coded equation may determine factor importance. The final coded factor equation is:

EE = +89.40 + 3.26A − 5.20B + 6.75AB − 9.07A^2^ − 1.82B^2^

PS = +148.80 − 16.24A − 49.67 + 14.25AB + 8.96A^2^ + 51.23B^2^

**Figure 1:**
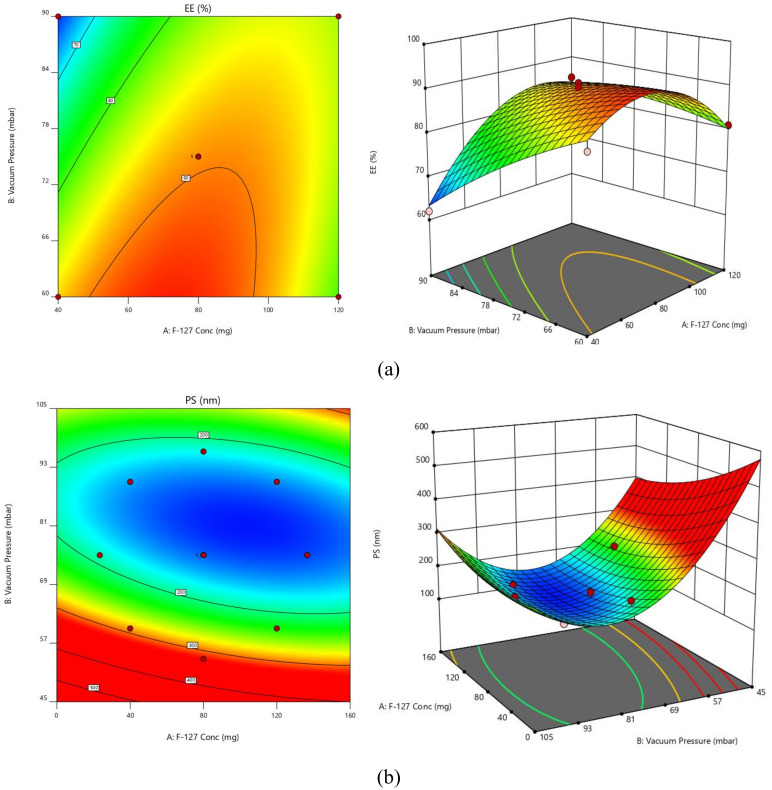
Contour plots and response surface graphs for (**a**) EE and (**b**) PS.

Any given concentration of the chosen factors can be predicted using any of the aforementioned equations. Additionally, factor coefficients aid in comparing their relative influence on the responses. The measured responses are depicted with these graphs, and contour plots and 3D response surface graphs (RSGs) are essential for illuminating the interaction and primary effect (Figure 1).

**Figure 2:**
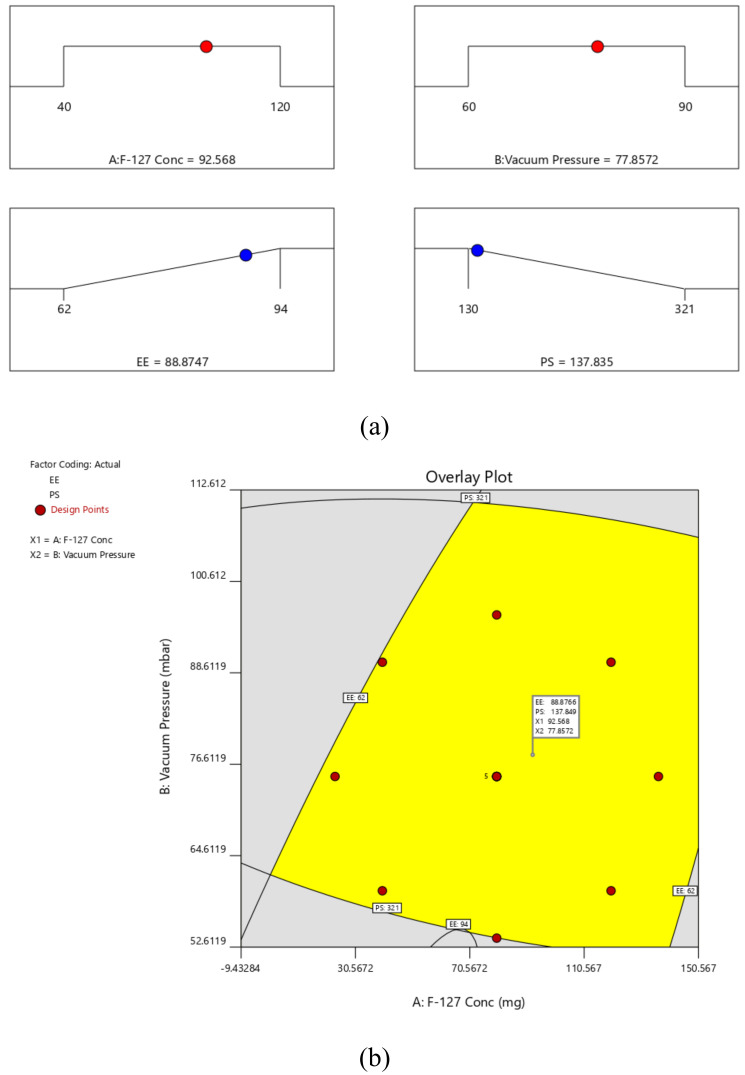
(**a**) Desirability and (**b**) overlay plot of optimized solution.

By using the desirability function (D), it was possible to optimize various models acquired through the experimental study. To produce the overlay graph, several constraints, including particle size, zeta potential, and PDI minimum, were specified for each response [29,30]. The design space included each and every one of the chosen variables. The maximum D value of 0.986 for the combined desirability plot of all the responses was reached at the best independent variable concentrations (Figure 2a), and the critical responses were superimposed in the contour plot (Figure 2b). Based on this desirable approach, the prerequisites of the optimum formulation can be achieved by a formulation having 92.568 mg of F-127 and 77.85 mbar vacuum pressure. As a result, EE of 88.8747% and PS of 0.137.835 can be obtained by applying these optimal concentrations. These concentrations allowed for the preparation and evaluation of the SIM-optimal NC’s formulation. To support the experimental design, the experimental results were compared with theoretical values. Relative error was discovered to be under 2%, which supports the design’s accuracy [52,53,54].

### 3.1. Surface Morphology

**Figure 3:**
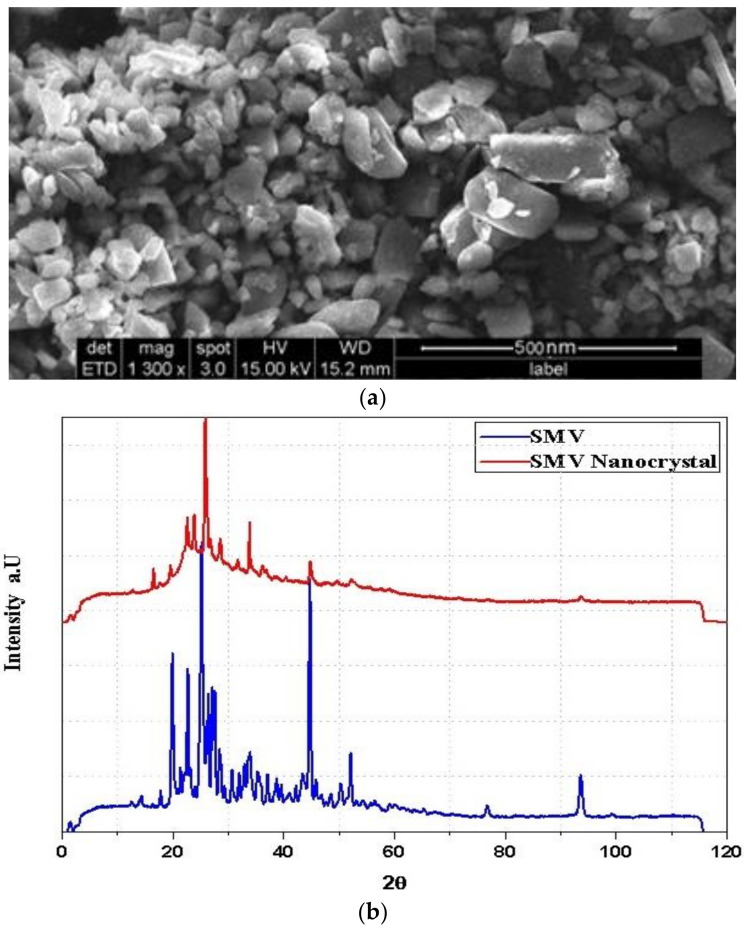
(**a**) SEM image of O-SIM-NC. (**b**) XRD patterns of SIM and SIM-NC.

The size and shape of O-SIM-NC were determined by SEM surface morphology characterization. With sizes ranging from 120 to 150 nm, naked SIM nanocrystals displayed a rod-like structure (Figure 3a) and kept their rod-like form when registering longer lengths. The relative length of nanocrystals is expected to decrease the renal clearance and increase plasma residence time [55]. Amorphous nature of the pure SIM and crystallinity of SIM-NC formulations are evident in the XRD graph (Figure 3b).

### 3.2. Drug Release Study

**Figure 4:**
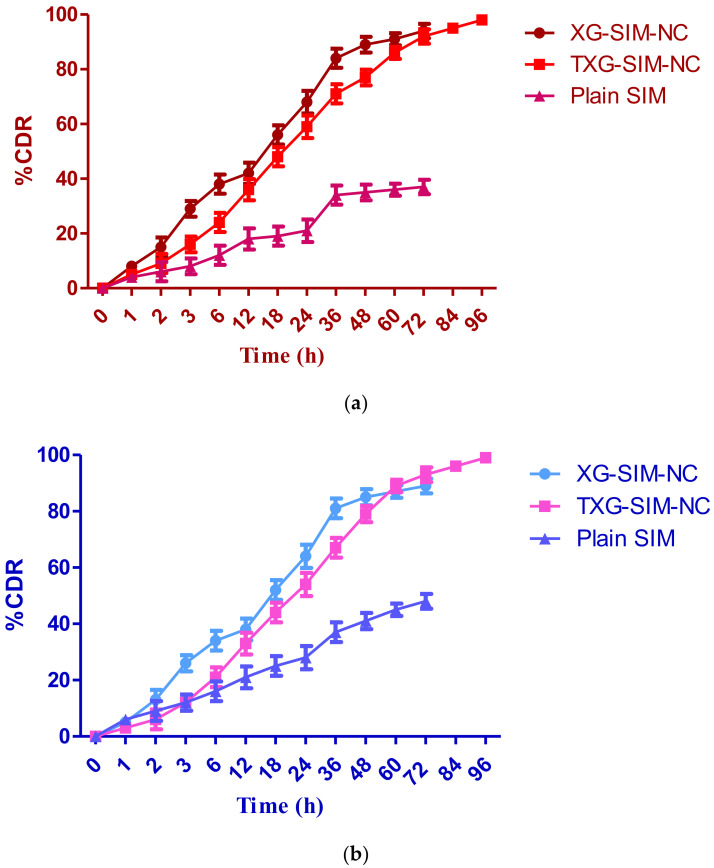
In vitro dissolution profile of optimized formulations of SIM-NC and plain SIM in (**a**) PBS—7.4 and (**b**) acidic buffer—pH 2.0.

To ascertain whether SIM would be released from nanocrystals prior to cellular absorption as a function of time, the in vitro drug release profile of SIM from XG-SIM-NC and TXG-SIM-NC was examined using two dissolution media of pH 2.0 and 7.4. Nanocrystals exhibit a significantly sustained release profile, as shown in Figure 4a,b. A partial SIM release was seen as a result of the solubility problems. After 12 h of rapid SIM release, steady-state SIM release was seen till the end of the trial. Beginning with the first 18 h, both formulations exhibit a rapid release of SIM. It contributes roughly 48–56% of the total amount of encapsulated SIM. This initial, accelerated release of SIM from NPs was mainly related to the presence of SIM at the NP surface, which allowed significant water diffusion across the liquid matrix and explained the accelerated drug release. In addition, a sustained phase with regular drug release is observed for the following 72 h. Although the pattern of release for the two profiles was comparable, there were differences in the amounts released. After 96 h, there had been a total of about 98.25% of the medicines released from the TXG-SIM-NC. A similar release profile was observed with pH 2.0 media, but the release of pure SIM was enhanced. This resulted from TXG’s gelling function, which regulated the drug release. Thiolation provides information about the arrangement of 3D gels and inter- and intrachain disulfide bonds (which could increase the matrix’s cross-linkage and cohesiveness), facilitating media diffusion.

### 3.3. Mucoadhesion Study

**Figure 5:**
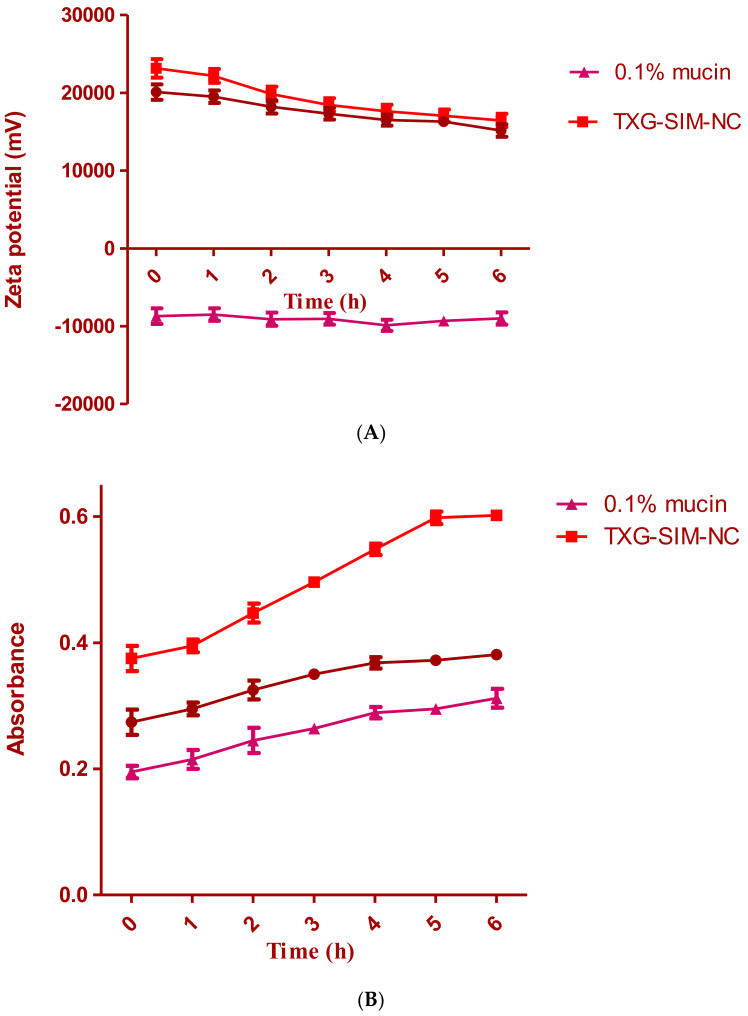
(**A**) The zeta potential of optimal formulations is estimated by turbidimetric assay during incubation in 0.1% aqueous mucin dispersion; (**B**) assessment of the interaction between SIM-NC and mucin dispersion.

The interaction between TXG-SIM-NC, XG-SIM-NC, and mucin was shown using two in vitro techniques. Zeta potential measurements of their dispersions were performed to gain insight into the mechanism of SIM-NC–mucin interaction. The results are displayed in Figure 5a. The most likely mucoadhesive mechanism is an electrostatic contact, which also explains why TXG-SIM-NC and XG-SIM-NC zeta potentials drop after being incubated with mucin. This might be the result of the interaction between the positively charged surface layer of SIM-NC and the negatively charged sialic groups of mucin. The ionic interaction between the negatively charged mucin particles and NPs, which occurred after 4 h of incubation with mucin, was responsible for the NC surface charge drop. Therefore, it may be said that ionic contact allowed the NCs and mucin to interact.

The absorbance of a 0.1% aqueous mucin dispersion at 650 nm was utilized as a reference for the turbidimetric investigation. The findings of the turbidimetric experiment are displayed in Figure 5b. The turbidity of optimal formulation dispersions was investigated to learn more about the nature of mucoadhesion. The mucin dispersions’ absorbance does not significantly deviate from 0.4. Changes in the turbidity of coated NC–mucin dispersions should not be attributed to particle mobility but rather as a sign of a potential interaction between NCs and mucin. Compared to XG-SIM-NC dispersion, TXG-SIM-NC dispersions had higher turbidity. The prior discussion of the TXG layer’s increased thickness and gel-forming ability around these particles may help to explain this phenomenon.

### 3.4. Cytotoxic Study

**Figure 6:**
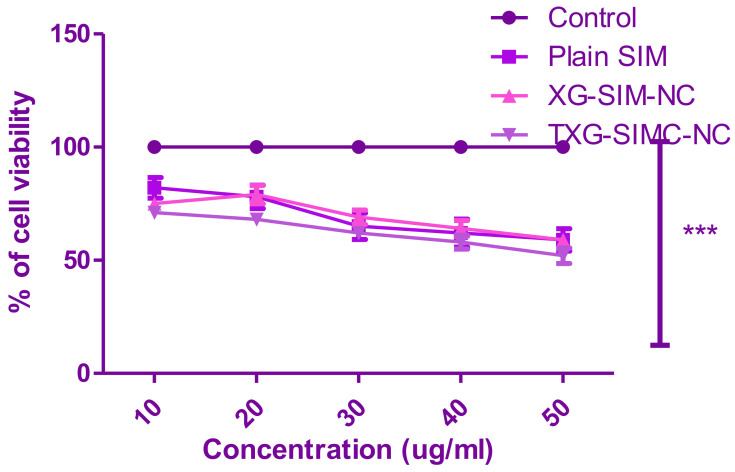
Effect of XG-SIM-NC, TXG-SIM-NC, plain SIM, and control (5-fluorouracil) on the percentage cell viability of HSC-3 cell lines. (The values indicated are the mean ± S.D, *n* = 9).

Following 72 h of treatment, the cytotoxicity of the improved formulations of SIM and plain SIM against HCS-3 cells was evaluated using the MTT test. Figure 6 contrasts the preparations’ cell viability percentage with the control group (normalized to 100). Cell viability tests were performed on two formulations (XG-SIM-NC and TXG-SIM-NC) and the control group at various concentrations (10–50 g/mL). This test demonstrates that all treatments reduced cell viability at the prescribed dose (in a dose-dependent manner). Since improved formulations’ observed percentage of cell viability was lower than that of the plain SIM, this indicated that the effect had potent cytotoxicity on HSC-3 cells.

### 3.5. Pharmacokinetic Study

A calibration curve was created using various concentrations of simvastatin in order to determine the unknown plasma drug concentration. Simvastatin’s nominal concentration and peak area were plotted to test the calibration curve’s linearity. Simvastatin was examined at eight different concentrations (0.02, 0.04, 0.06, 0.08, 0.1, 0.12, 0.14, and 0.16 µg/mL) for the linearity experiments. Over the concentration range under study, the peak area response was linear. The correlation coefficient, or “r2,” was discovered to be 0.999. After a single dosage of the test, samples were administered, an unknown plasma drug concentration was obtained, and the pharmacokinetic data were effectively determined using the HPLC interpolation methodology. A calibration curve was used to determine the unknown concentration. Simvastatin’s mean plasma concentration as a function of time has been displayed, and Table 5 compares the pharmacokinetic characteristics of the SIM suspension and improved formulations.

**Figure 7:**
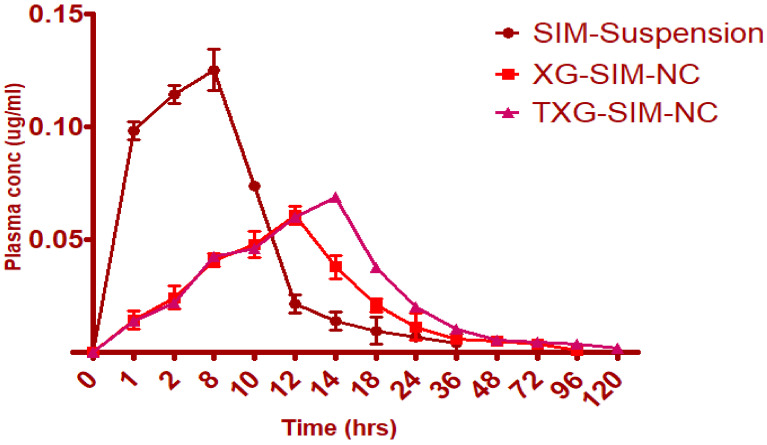
Comparison of pharmacokinetic profiles of SIM suspension, XG-SIM-NC, and TXG-SIM-NC.

When compared to XG-SIM-NC, it was found that TXG-SIM-NC controlled the release as well as pharmacokinetic characteristics, as shown in Table 4. The pharmacokinetic characteristics of the SIM suspension and mucoadhesive NC drug delivery systems significantly differed. The maximum time for TXG-SIM-NC was found to be 14 h, and the maximum time for XG-SIM-NC was 12 h. As anticipated, ordinary SIM suspension reached Cmax (0.1254) in only 8 h (Figure 7). TXG-SIM-NC demonstrated a somewhat higher Cmax than XG-SIM-NC. For the SIM suspension, XG-SIM-NC, the area under the curve (AUC_0-α_) was determined to be 84.6528 µg/mL/h, 1453.0478 µg/mL/h, and 1847.0654 µg/mL/h, respectively. It was discovered that the mean residence time (MRT) for the thiol formulation was more significant at 98 h, which is attributable to the improved mucoadhesive capability of XG. According to the in vivo pharmacokinetics data, SIM-NC formulations showed higher AUC_0-α_, Tmax, and MRT with lower Cmax values when compared to plain SIM. It has been determined that XG-SIM-NC and TXG-SIM-NC exhibit an increase in bioavailability of approximately 17.16% and 21.82% compared to the SIM suspension formulation as a benchmark. Finally, the pharmacokinetic profile can be correlated with in vitro drug release pattern. The in vitro drug release profile shows maximum drug release by the end of 36 h followed by a very slow phase of drug release. By the end of 24 h, more than 70% of the drug has been released. SIM absorption in in vivo conditions can be summarized as follows: Cmax was reached at the end of 14 h, but the drug concentration was maintained within the therapeutic range till the end of 96 h. Till the end of 72 h, the pattern of SIM release and absorption was found to be similar, the slight changes in the profiles can be attributed the presence of mucus later during in vivo studies.

## 4. Conclusions

We reported here that a simple three-step methodology could formulate NCs of SIM. In developing the SIM-NCs, RSM, in association with various statistical estimations, has been applied to optimize the various process variables in the formulation. Based on this desirability approach, a formulation containing 92.568 mg of F-127 and 77.85 mbar vacuum pressure can accomplish the prerequisites of the optimized formulation. They resulted in EE of 88.8747% and PS of 0.137.83. Final NCs were made into suspensions using XG and TXG. The formulation made with TXG shows its excellent properties in terms of drug release and mucoadhesion potential. The mucoadhesion property further enhanced the cytotoxic nature of the formulated SIM-NC. The relative bioavailability of TXG-SIM-NC was about 21.82%, in contrast with the plain SIM suspension. Furthermore, the optimized method is believed to be suitable for formulating other hydrophobic drugs. Lastly, the NCs can be further modified for targeted delivery of anticancer drugs to increase their therapeutic efficacy.

## Figures and Tables

**Table 1:** Experimental plan for central composite design (CCD) in terms of actual and coded values.

Factors/Independent Variables	Levels					Responses/Dependent Variables	Constraints
	−1.414	−1	0	+1	+1.414		
F-127 Conc.—X_1_	23.4315	40	80	120	136.569	EE	Maximum
Vacuum Pressure—X_2_	53.7868	60	75	90	96.2132	PS	Minimum

**Table 2:** Experimental runs projected and their observed responses.

		Factor 1	Factor 2	Response 1	Response 2
Std	Run	A: F-127 Conc	B: Vacuum Pressure	EE	PS
		mg	mbar	%	nm
3	1	40	90	62	147
8	2	80	96.2132	78	185
10	3	80	75	91	165
4	4	120	90	85	165
9	5	80	75	88	144
11	6	80	75	89	148
5	7	23.4315	75	69	207
7	8	80	53.7868	94	321
6	9	136.569	75	74	130
13	10	80	75	89	145
1	11	40	60	85	278
12	12	80	75	90	142
2	13	120	60	81	239

**Table 3:** Model statistical summary.

Response	Models	*R* ^2^	Adju. *R*^2^	Pred. *R*^2^	Adequate Precision	Sequential *p*-Value	Remarks
EE	Linear	0.2780	0.1336	−0.3744	----	0.1962	
	2 FI	0.4460	0.2613	−0.2797	23.1661	0.1329	
	Quadratic	0.9768	0.9603	0.8618	---	<0.0001	Suggested
	Cubic	0.9947	0.9874	0.9630	---	0.0245	
PS	Linear	0.5163	0.4196	0.1086	---	0.0265	
	2 FI	0.5355	0.3807	−0.0348	---	0.5570	
	Quadratic	0.9679	0.9450	0.8174	18.7863	<0.0001	Suggested
	Cubic	0.9912	0.9789	0.9501	---	0.0392	

**Table 4:** Analysis of variance (ANOVA) results.

	Intercept	A	B	AB	A^2^	B^2^
EE	89.4	3.25888	−5.20343	6.75	−9.075	−1.825
*p*-values		0.0018	0.0001	0.0002	<0.0001	0.0386
PS	148.8	−16.2368	−49.6666	14.25	8.975	51.225
*p*-values		0.0132	<0.0001	0.0799	0.1329	<0.0001

**Table 5:** Comparison of pharmacokinetic properties of test samples.

Parameter	SIM Suspension	XG-SIM-NC	TXG-SIM-NC
T_max_ (h)	8	12	14
C_max_ (µg/mL)	0.1254	0.061	0.069
AUC_0-∞_ (µg/mL h)	84.6528	1453.0478	1847.0654
MRT_0-v_ (h)	23	72	96
F_rel_	--	17.16% enhanced bioavailability	21.82% enhanced bioavailability

## Data Availability

Not applicable.

## Supplementary Materials

The following supporting information can be downloaded at: https://www.mdpi.com/article/10.3390/polym14235184/s1, Figure S1: The normal plot of residuals and residuals vs Run for EE and PS.
