# Supplementary figures and images for: Response Surface Methodology (RSM) Powered Formulation Development, Optimization and Evaluation of Thiolated Based Mucoadhesive Nanocrystals for Local Delivery of Simvastatin
Source: Polymers (Basel). 2022 Nov 28;14(23):5184. doi: 10.3390/polym14235184 (PMC9737842; doi:10.3390/polym14235184)

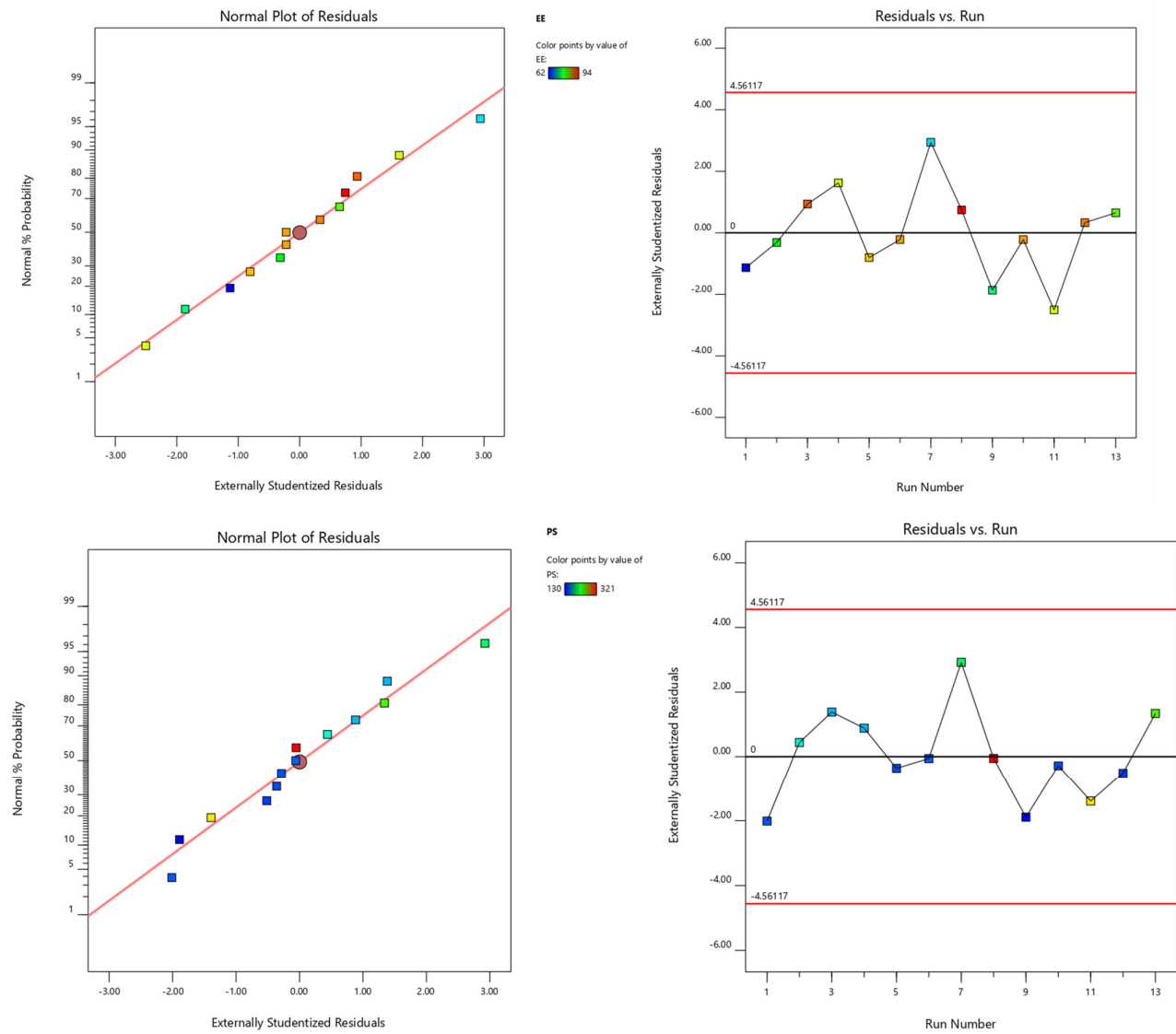

**Figure S1.** The normal plot of residuals and residuals vs Run for EE and PS.

Supplement: Supplementary file 1 [file polymers-14-05184-s001.zip › polymers-1958992-supplementary.pdf]
